# Systematic review of distributed practice and retrieval practice in health professions education

**DOI:** 10.1007/s10459-023-10274-3

**Published:** 2023-08-24

**Authors:** Emma Trumble, Jason Lodge, Allison Mandrusiak, Roma Forbes

**Affiliations:** 1 School of Education, The University of Queensland, Queensland, Australia; 2 School of Health and Rehabilitation Sciences, The University of Queensland, Queensland, Australia

**Keywords:** Health professions education, Distributed practice, Retrieval practice

## Abstract

To determine the effect of distributed practice (spacing out of study over time) and retrieval practice (recalling information from memory) on academic grades in health professions education and to summarise a range of interventional variables that may affect study outcomes. A systematic search of seven databases in November 2022 which were screened according to predefined inclusion criteria. The Medical Education Research Study Quality Instrument (MERSQI) and Newcastle-Ottawa Scale-Education (NOS-E) were used to critically appraise eligible articles. A summary of interventional variables includes article content type, strategy type, assessment type and delay and statistical significance. Of 1818 records retrieved, 56 were eligible for inclusion and included a total of 63 experiments. Of these studies, 43 demonstrated significant benefits of distributed practice and/or retrieval practice over control and comparison groups. Included studies averaged 12.23 out of 18 on the MERSQI and averaged 4.55 out of 6 on the NOS-E. Study designs were heterogeneous with a variety of interventions, comparison groups and assessment types. Distributed practice and retrieval practice are effective at improving academic grades in health professions education. Future study quality can be improved by validating the assessment instruments, to demonstrate the reliability of outcome measures. Increasing the number of institutions included in future studies may improve the diversity of represented study participants and may enhance study quality. Future studies should consider measuring and reporting time on task which may clarify the effectiveness of distributed practice and retrieval practice. The stakes of the assessments, which may affect student motivation and therefore outcomes, should also be considered.

## Introduction

Health professions education (such as medicine, physiotherapy, and clinical psychology) covers a large amount of theoretical and practical content over a broad range of subjects, to prepare students for entering the workplace. Distributed practice (spaced practice) is spacing out study over time as opposed to massing (or cramming) study. Retrieval practice is the act of recalling information from memory, such as using practice tests. Both strategies have been reported as effective at improving knowledge retention in a number of contexts and are thus considered to benefit health professions education (Dunlosky et al., 2013). These strategies are also considered ‘desirable difficulties’, coined to represent study strategies that feel challenging but are often more effective than those that feel easy (Bjork & Bjork, 2011). Previous research in health professions education demonstrates that students and educators often hold misconceptions about what are effective study strategies, and commonly use strategies that are considered less effective (Piza et al., 2019). Even with a clear concept of effective study strategies, students during a unit of learning will often revert to less effective strategies than originally intended (Blasiman et al., 2017). Exploring the effectiveness of distributed practice and retrieval practice in health professions education is therefore indicated to help guide students and educators.

Distributed practice in previous research is often compared to no intervention, massed study, or varying the inter-study interval (ISI). ISI is the interlude separating different study sessions, and consists of three main types: expanding, contracting and equal. Expanding schedules refer to the gradual increase in ISIs, contracting schedules are the gradual decrease in ISIs and equal schedules are equally spaced ISIs, with research demonstrating varying effectiveness (Gerbier et al., 2015; Karpicke & Roediger, 2007, 2010; Küpper-Tetzel et al., 2014). The overall length of an ISI also affects learning outcomes, with increasing ISIs up to 29 days demonstrating improved long term memory outcomes when compared to shorter ISIs (Cepeda et al., 2006; Rohrer, 2015). Furthermore, as the retrieval interval increases, concurrently increasing the ISIs improves outcomes compared to shorter ISIs (Cepeda et al., 2008).

Retrieval practice includes three main types, each varying in cognitive load: recognition, cued recall, and free recall. Recognition questions, such as multiple-choice, allow students to select an answer that they recognise but may have been unable to recall without the suggestion. Cued recall questions refer to fill-in-the-blank and short-answer questions which increase cognitive demand, and free recall is considered the most cognitively demanding, as no question cue, or answer suggestion is provided (Adesope et al., 2017). Retrieval practice that increases cognitive demand, correlates with improves assessment scores (Adesope et al., 2017; Rowland, 2014) and retrieval practice questions that are identical to assessment questions are reported to be more effective than non-identical retrieval practice questions (Veltre et al., 2015).

The comparison groups for retrieval practice can include no study or normal class, restudying (rereading or rewatching content), concept mapping, or comparisons between varying types of retrieval practice, with retrieval practice generally demonstrating superior outcomes in all comparisons (Adesope et al., 2017). Including feedback with retrieval practice has shown mixed results, with positive outcomes found in lab-based studies, but null effect in classroom-based studies (Adesope et al., 2017). Further, some studies showed a reduced effect when feedback was added to retrieval practice (Kliegl et al., 2019; Racsmány et al., 2020). Longer retrieval intervals, the interval between practice and final assessment, favours retrieval practice over restudy (Rowland, 2014). One specific use of retrieval practice is pre-questions, which is the retrieval of information that has yet to be covered, and may also enhance retention of that material (Little & Bjork, 2016; Richland et al., 2009).

Time on task is also an important variable to track in comparison trials. Increasing time on task has shown a strong correlation with improved academic grades (Chang et al., 2014). Therefore, this could be a confounding factor if distributed practice or retrieval practice time on task does not equal that of the comparison or control group. Controlling for time on task in trials will reduce the risk of this factor confounding results.

The stakes of an assessment may also be relevant, defined as formative assessments (or no-stakes assessments) and summative assessments which can be low-stakes (low weighting or grade) or high-stakes, such as exams that must be passed to complete a unit. Mixed outcomes have been found when increasing the stakes of assessments. High stakes may increase the motivation to engage with the learning strategy, thereby improving outcomes (Phelps, 2012). However, increased stakes may induce test anxiety, thereby reducing final performance (Hinze & Rapp, 2014). Learning setting is also important, with interventions that are applied to assessments and coursework relevant to educators, whereas interventions applied to self-directed learning, such as homework are also applicable to students.

How distributed practice and retrieval practice are implemented may affect the outcome. Therefore, this review also summarises key implementation variables, including type of retrieval practice and distributed practice, type of comparison group, the inclusion of feedback with retrieval practice, the retrieval interval, time on task and the stakes of an assessment. Included in this review is also a critical appraisal of the methodology quality of studies and therefore the strength of the results. No current systematic review appraises the distributed practice and retrieval practice literature in a health professions education context, however, there has been related work with a scoping review of spaced learning in health professions education (Versteeg et al., 2020), a systematic review of instructional design in simulation-based education (Cook et al., 2013) and a review of brain-aware teaching strategies for health professions education (Ghanbari et al., 2019).

The purpose of this review is to determine the effect of distributed practice and retrieval practice on academic grades in health professions education. This review will highlight directions for future research and guide educators and students towards more effective learning strategies to assist in improving knowledge acquisition.

## Methods

A systematic review method was applied according to the PRISMA guidelines to answer the review question: Are distributed practice and retrieval practice effective learning strategies at improving academic grades in health professions education?

The inclusion criteria are outlined in Table 1 and articles were only included from peer reviewed journals. Both control and comparison studies were included in this review, however case series were excluded. Studies were excluded if the intervention, control, or comparison groups did not have equivalent outcome measures. Laboratory studies were excluded to improve the applicability of the research to health professions education. Content relevant to tertiary healthcare programs was searched via healthcare professions, which are included in the search criteria listed below. These were further screened for applicability, with graduate programs and studies that included non-clinical content, such as cognitive psychology studies excluded. There were no exclusion criteria for comparison groups, therefore both control groups and a variety of comparison groups were included in this review. Studies were excluded if the only outcome measure was students’ subjective rating of their performance, as this is often an inaccurate judgement of learning (Dunlosky & Rawson, 2012). Academic grades were therefore a required outcome measure for inclusion, despite satisfaction, judgement of learning and engagement also benefitting from both distributed practice and retrieval practice (Browne, 2019; Bruckel et al., 2016; Karpicke, 2009; Son & Metcalfe, 2000).

**Table 1:** Table 1

Population	Participants in tertiary level health professions education. Representative educational context studies.
Study design	Randomised controlled trials, controlled or comparative trials.
Outcomes	Grade point average, academic results in theory or practical exams, quizzes, assignments, and supervisor graded clinical placements
Intervention	Distributed practice and/or retrieval practice.
Language	English

### Search strategy

#### Identification

The population and intervention inclusion criterion were used to create search terms, including alternate terms such as spaced practice for distributed practice. This method was applied to the databases of EBSCOhost (Education Source, CINAHL Complete, ERIC, MEDLINE Complete, Psychology and Behavioral Sciences Collection), Web of Science, and Scopus. Search terms: (health OR physiotherap* OR “physical therap*” OR “allied health” OR pharmacy OR medic* OR nursing OR “occupational therap*” OR “speech patholog*” OR dentist* OR psycholog*) AND (student* OR undergrad* OR postgrad* OR tertiary OR universit*) AND (“retrieval practice” OR “retrieval-based practice” OR “spaced practice” OR “distributed practice”) in November 2022. Search mode: EBSCOhost ‘find all my search terms’, Web of Science ‘TOPIC’, Scopus ‘article title, abstract and keywords’.

#### Screening, eligibility, and inclusion

**Figure 1:**
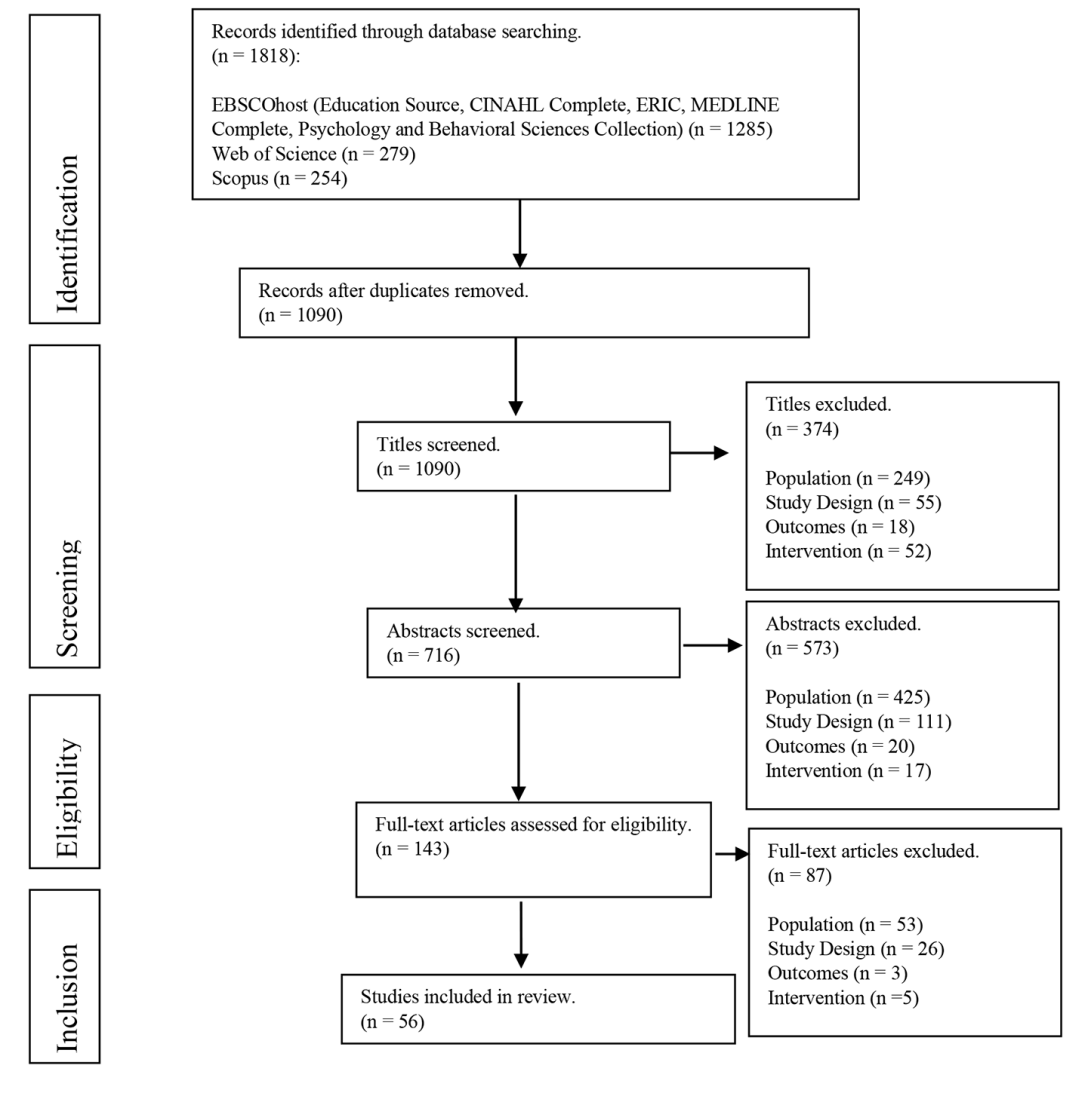
Figure 1

After removal of duplicate articles, the remaining articles were screened for eligibility by title, then abstract and finally the full article against the inclusion and exclusion criteria. The results of this screening process are displayed in Fig. 1, with the most common reasons for exclusion being non-tertiary health professions education, such as other non-clinical healthcare disciplines, qualified healthcare professions education, or clinical healthcare patient populations.

#### Critical appraisal

The Medical Education Research Study Quality Instrument (MERSQI) and Newcastle-Ottawa Scale-Education (NOS-E) were used to critically appraise the eligible articles (Cook & Reed, 2015). One review of these appraisal methods suggests that whilst the MERSQI focuses on more objective design issues, the NOS-E is more subjective, but covers more information on the implications of study design. They therefore complement each other when used together (Cook & Reed, 2015). See ‘Table 1’ of the article by Cook, David A. MD, MHPE and Reed, Darcy A. MD, MPH for further information of the criteria definitions and scoring system of the MERQI and NOS-E (Cook & Reed, 2015).

#### Summary of articles

The summary will also highlight the key variables described in the introduction. Statistical significance will only briefly spotlight significant findings and for studies that compare multiple timepoints, the statistical significance summary will focus on the longest retrieval interval.

## Results

The MERSQI score for each included study is provided in Table 2 and NOS-E score in Table 3. The studies’ variables, and statistical significance are summarised in Table 4.

**Table 2:** Table 2

Study	Study design	Sampling	Type of Data	Validity of Evaluation Instrument	Data Analysis	Outcomes	Total
(Amabile et al., 2021)	2	2	3	0	3	1.5	11.5
(Anders et al., 2022)	2	1	3	1	3	1.5	11.5
(Biwer et al., 2022)	2	2	3	0	3	1.5	11.5
(Blasiman, 2017)	2	2	3	0	3	1.5	11.5
(Breckwoldt et al., 2016)	2	2	3	1	3	1.5	12.5
(Brown-Kramer, 2021)	2	2	3	0	3	1.5	11.5
(Burdo & O’Dwyer, 2015)	2	2	3	0	3	1.5	11.5
(Cadaret & Yates, 2018)	3	2	3	0	3	1.5	12.5
(Carpenter et al., 2016)	3	2	3	0	3	1.5	12.5
(Carpenter et al., 2018)	3	2	3	0	3	1.5	12.5
(Cecilio-Fernandes et al., 2018)	2	2	3	0	3	1.5	11.5
(Dobson & Linderholm, 2015a, b)	3	2	3	0	3	1.5	12.5
(Dobson et al., 2018)	3	2	3	0	3	1.5	12.5
(Dobson, 2011)	3	2	3	0	3	1.5	12.5
(Dobson, 2012)	3	2	3	0	3	1.5	12.5
(Dobson, 2013)	3	1.5	3	0	3	1.5	12
(J. L. Dobson and Linderholm, 2015a, b)	3	2	3	0	3	1.5	12.5
(Dobson et al., 2019)	3	2	3	0	3	1.5	12.5
(Dobson et al., 2015)	3	2	3	0	3	1.5	12.5
(Dobson et al., 2017)	3	2	3	0	3	1.5	12.5
(Ernst et al., 2014)	3	2	3	0	3	1.5	12.5
(Fendos, 2020)	2	2	3	1	3	1.5	12.5
(Francis et al., 2020)	2	2	3	1	3	1.5	12.5
(Glass et al., 2013)	2	1	3	0	3	1.5	10.5
(Gopalan et al., 2020)	2	2	3	1	3	1.5	12.5
(Gurung & Burns, 2019)	2	3	3	1	3	1.5	13.5
(Hernick, 2015)	2	2	3	0	3	1.5	11.5
(Higham et al., 2022)	3	2	3	0	3	1.5	12.5
(Iwamoto et al., 2017)	3	2	3	0	2	1.5	11.5
(Janes et al., 2020)	3	2	3	1	3	1.5	13.5
(Kerdijk et al., 2015)	3	2	3	0	3	1.5	12.5
(Keus et al., 2019)	2	2	3	0	2	1.5	10.5
(LaDisa & Biesboer, 2017)	2	2	3	0	3	1.5	11.5
(Lawson, 2022)	3	2	3	0	3	1.5	12.5
(Linderholm et al., 2016)	3	2	3	1	3	1.5	13.5
(Logan et al., 1975)	3	2	3	1	2	1.5	12.5
(Messineo et al., 2015)	3	2	3	0	3	1.5	12.5
(Miller & Srimaneerungroj, 2022)	2	2	3	0	3	1.5	11.5
(Moore & Chalk, 2012)	2	2	3	0	3	1.5	11.5
(Nevid et al., 2016)	3	2	3	0	3	1.5	12.5
(Oermann et al., 2022a, b)	3	2.5	3	1	3	1.5	14
(Oermann et al., 2022a, b)	3	2	3	1	3	1.5	13.5
(Opre et al., 2022)	2	2	3	0	3	1.5	11.5
(Osterhage et al., 2019)	2	2	3	0	3	1.5	11.5
(Palmen et al., 2015)	2	2	3	0	3	1.5	11.5
(Palmer et al., 2019)	3	2	3	0	3	1.5	12.5
(Poorthuis & van Dijk, 2021)	2	2	3	0	3	1.5	11.5
(Schmidmaier et al., 2011)	3	2	3	1	3	1.5	13.5
(Schneider et al., 2019)	3	2	3	1	3	1.5	13.5
(Sennhenn-Kirchner et al., 2018)	3	2	3	1	3	1.5	13.5
(Shobe, 2022)	2	1	3	0	3	1.5	10.5
(Terenyi et al., 2018)	3	1	3	0	3	1.5	11.5
(Terenyi et al., 2019)	3	1	3	0	3	1.5	11.5
(Timmer et al., 2020)	3	1.5	3	2	3	1.5	14
(Trumbo et al., 2016) Exp 1	2	1	3	1	3	1.5	11.5
(Trumbo et al., 2016) Exp 2	3	1.5	3	1	3	1.5	13
(Trumbo et al., 2016) Exp 3	3	2	3	1	3	1.5	13.5
(Wong, 2022)	2	1.5	3	0	3	1.5	11
**Average Score**	**2.57**	**1.89**	**3**	**0.33**	**2.95**	**1.5**	**12.23**

**Table 3:** Table 3

Study	Representativeness of Intervention Group	Selection of Comparison Group	Comparability of Comparison Group	Study Retention	Blinding of Assessment	Total
(Amabile et al., 2021)	0	0	2	1	1	4
(Anders et al., 2022)	0	1	0	0	1	2
(Biwer et al., 2022)	1	1	1	1	1	5
(Blasiman, 2017)	1	1	0	0	1	3
(Breckwoldt et al., 2016)	1	1	0	1	0	3
(Brown-Kramer, 2021)	1	1	0	1	0	3
(Burdo & O’Dwyer, 2015)	1	1	1	1	1	5
(Cadaret & Yates, 2018)	1	1	1	1	1	5
(Carpenter et al., 2016)	1	1	1	1	1	5
(Carpenter et al., 2018)	1	1	1	1	1	5
(Cecilio-Fernandes et al., 2018)	1	1	0	1	1	4
(Dobson & Linderholm, 2015a, b)	1	1	1	1	1	5
(Dobson et al., 2018)	1	1	1	1	1	5
(Dobson, 2011)	1	1	2	1	1	6
(Dobson, 2012)	1	1	2	1	1	6
(Dobson, 2013)	0	1	2	0	1	4
(J. L. Dobson and Linderholm, 2015a, b)	1	1	2*	1	1	6
(Dobson et al., 2019)	1	1	2*	1	1	6
(Dobson et al., 2015)	1	1	2*	1	1	6
(Dobson et al., 2017)	1	1	2*	1	1	6
(Ernst et al., 2014)	1	1	1	1	1	5
(Fendos, 2020)	1	1	2*	1	1	6
(Francis et al., 2020)	1	1	2*	1	1	6
(Glass et al., 2013)	0	1	2*	0	1	4
(Gopalan et al., 2020)	1	1	0	1	0	3
(Gurung & Burns, 2019)	1	0	2	1	1	5
(Hernick, 2015)	1	1	2	1	0	5
(Higham et al., 2022)	1	1	2*	1	1	6
(Iwamoto et al., 2017)	1	1	0	0	0	2
(Janes et al., 2020)	1	1	2*	1	1	6
(Kerdijk et al., 2015)	0	1	2	1	1	5
(Keus et al., 2019)	1	0	1	1	1	4
(LaDisa & Biesboer, 2017)	1	0	0	1	1	3
(Lawson, 2022)	1	1	2*	1	1	6
(Linderholm et al., 2016)	0	1	1	1	1	4
(Logan et al., 1975)	0	1	2	1	0	4
(Messineo et al., 2015)	0	1	2	1	1	5
(Miller & Srimaneerungroj, 2022)	1	1	2*	1	1	6
(Moore & Chalk, 2012)	0	1	0	1	0	2
(Nevid et al., 2016)	0	1	2	1	1	5
(Oermann et al., 2022a, b)	1	1	2	0	1	5
(Oermann et al., 2022a, b)	1	1	2	0	1	5
(Opre et al., 2022)	0	1	1	0	1	3
(Osterhage et al., 2019)	0	1	0	1	1	3
(Palmen et al., 2015)	0	1	2*	1	0	4
(Palmer et al., 2019)	0	1	2*	1	1	5
(Poorthuis & van Dijk, 2021)	1	1	0	1	1	4
(Schmidmaier et al., 2011)	0	1	2	1	1	5
(Schneider et al., 2019)	1	1	2	1	1	6
(Sennhenn-Kirchner et al., 2018)	1	1	2	1	1	6
(Shobe, 2022)	0	1	1	0	1	3
(Terenyi et al., 2018)	1	1	2*	0	1	5
(Terenyi et al., 2019)	1	1	2*	0	1	5
(Timmer et al., 2020)	0	1	2	0	1	4
(Trumbo et al., 2016) Exp 1	1	1	0	0	1	3
(Trumbo et al., 2016) Exp 2	1	1	2*	0	1	5
(Trumbo et al., 2016) Exp 3	1	1	2*	1	1	6
(Wong, 2022)	0	0	0	1	0	1
**Average score**	**0.69**	**0.91**	**1.34**	**0.76**	**0.84**	**4.55**

Studies denoted with an * are within-subject designs

**Table 4:** Table 4

Study	Content	Comparison Groups	Assessment
(Amabile et al., 2021)	Anatomy (course)	A: Lectures, 45 h and Labs, 90 h (Distributed over 2 × 15-week semesters) B: Lectures, 45 h and Labs, 90 h (Massed over a 10-week semester)	REC (month 18) (NGR)
(Anders et al., 2022)	Physiology (portion of course)	A: Lectures, audience response questions (optional participation) and practice examinations (optional homework) ^α^ B: Lectures, historical control (No TOT recorded) (no feedback recorded)	Retrieval type not reported (End of course, no retrieval interval reported) (FA or SA − 10%)
(Biwer et al., 2022)	Pathophysiology (pre-course)	A: Education about and practice of retrieval practice and distributed practice (class, weeks 2–4, reminders weeks 5–18) ^α^ B: Normal class (No TOT recorded)	REC (exams week 8, 13 and 18) (SA)
(Blasiman, 2017)	Introductory psychology (portion of course)	A: CR in group previous lectures content (CR) ^α^ (class, weeks 1–5, 5–10 min) (feedback) B: Normal Class	REC (exam 2: week 6) (SA) (not identical)
(Breckwoldt et al., 2016)	Emergency medicine (intensive course)	A: Lectures, 26 h total (Distributed over 4.5 days) ^α^ B: Lectures, 26 h total (Massed over 3 days)	A: CR (day 5) B: CR (day 5 or 8 or 13) (FA, immediately prior to SA)
(Brown-Kramer, 2021) Hypothesis 2	Introductory psychology (course)	A: Assignment (practice testing) ^α^ B: Assignment (distributed practice) C: Assignment (rereading) D: Assignment (forming mental images) (Essay assigned week 5, draft week 10, feedback by week 12) (No TOT recorded)	REC (exam 3 week 13 and Exam 4 week 16) (NGR)
Hypothesis 3	Introductory psychology (course)	A: Assignment options as above ^α^ B: Historical control (assignment of one article, no application section) (No TOT recorded)	REC (exam 3 week 13 and Exam 4 week 16) (NGR)
(Burdo & O’Dwyer, 2015)	Introductory psychology (course)	A: REC and CR generation and retrieval in group (class, weeks 1–12, 45-minutes) (feedback) ^α^ B: Concept maps in groups (class, weeks 1–12, 45-minutes) (feedback) C: No additional study	Primarily REC and CR (four module and one final exam) (SA: 4 highest counted, 20% each) (not identical)
(Cadaret & Yates, 2018)	Veterinary anatomy and physiology (course)	REC and CR (homework weekly, day 5 after class) (feedback) ^α^ REC and CR (homework weekly, day 1 after class) (feedback) (No TOT recorded)	REC and CR (week 13) (SA) (not identical)
(Carpenter et al., 2016)	Introductory biology (20 min in class)	Recall definitions and draw diagram ^α^ Recall definitions and label diagram ^α^ Copy definitions and draw diagram Copy definitions and label diagram (Class) (no TOT recorded) (feedback)	REC (day 5) (FA) (not identical)
(Carpenter et al., 2018)	Introductory psychology (course)	A: CR one pre-question (no feedback) and two CR end of class (one identical, one new) (classes weekly) (feedback) ^α^ B: Two CR end of class (classes weekly, less TOT) (feedback) ^α^ C: Control questions not practiced	CR (day 7) (NGR) (identical)
(Cecilio-Fernandes et al., 2018)	Oncology (course/content)	A: Content distributed over 3 years B: Content massed in first semester of third year ^α^ (Classes) (no TOT recorded)	REC (four in third year) (SA – must pass three)
(Dobson & Linderholm, 2015a, b) Phase 1	Anatomy and physiology (class)	A: Read, free recall, read ^α^ B: Read, read take notes C: Read, read, read (Class, TOT 4 × 5-7 min, day 0 and 5)	REC (day 7) (NGR) (not identical)
Phase 2	Anatomy and physiology (portion of course)	A: Education on results and benefits of retrieval practice ^α^ B: Historical classes (Classes) (no TOT recorded)	Retrieval type not reported (third, fourth and fifth exams) (NGR)
(Dobson et al., 2018)	Physiology (class)	A: Text read, read, read, read (day 1, TOT 4 × 4 min) B: Text read, free recall, read, free recall (day 1, TOT 4 × 4 min) ^α^ C: Article read, read, read, read (day 5, TOT 4 × 6.25 min) D: Article read, free recall, read, free recall (day 5, TOT 4 × 6.25 min) ^α^ (Classes)	Text A&B:CR (day 7) Article C&D: FR (day 7) (NGR) (partially identical)
(Dobson, 2011)	Physiology (class)	A: REC and CR (days 1, 2 and 3 equal distribution) B: REC and CR (days 0, 1 and 3 expanding distribution) ^α^ (Homework) (no feedback) (no TOT recorded)	REC and CR (day 10) (FA) (identical)
(Dobson, 2012)	Physiology (class)	A: REC and CR (days 1, 10 and 20 equal distribution) B: REC and CR (days 8, 15 and 22 equal distribution) C: REC and CR (days 1, 6 and 16 expanding distribution) ^α^ D: REC and CR (days 2, 7 and 17 expanding distribution) ^α^ (Homework) (no feedback) (no TOT recorded)	REC and CR (day 30) (FA) (identical)
(Dobson, 2013) Retrieval practice subset	Anatomy and physiology (course)	A: REC (homework, no TOT recorded, most weeks 5–15) (no feedback) ^α^ B: No retrieval practice	REC (week 16) (FA) (not identical, similar)
Distributed practice subset	Anatomy and physiology (course)	A: REC (expanding distribution) B: REC (equal distribution) (Homework weeks 5–15, no TOT recorded)	REC (week 16) (FA) (not identical, similar)
(J. L. Dobson and Linderholm, 2015a, b)	Anatomy (class)	A: Read, SAQ, read, SAQ ^α^ B: Read, FIB, read, FIB C: Read, read, read, read (Class) (TOT 4 × 3 min)	SAQ (day 7) (FA) (identical)
(Dobson et al., 2019)	Physiology (class)	A: Read, FR, read, FR ^α^ B: Read, JOL, read, JOL C: Read, read, read, read (Class) (TOT 4 × 4.5 min)	CR (day 7) (FA) (not identical)
(Dobson et al., 2015)	Anatomy (class)	A: Read, CR, read, CR ^α^ B: Read, read, read, read (Class) (TOT 4 × 2 min)	CR (day 7 and 21) (FA) (identical)
(Dobson et al., 2017)	Anatomy (class)	A: Read, CR, read, CR (day 0, 5 and 7) ^α^ B: Read, read, read, read (day 0, 5 and 7) C: Read, CR, read, CR, read CR (day 5 and 7) D: Read, read, read, read, read, read (day 5 and 7) E: Read, CR, read, CR, read, CR, read, CR, read, CR, read, CR (day 7) (Class) (TOT 4 × 4 min)	CR (day 7, 28) (FA) (identical)
(Ernst et al., 2014)	Paediatric intubation (intensive course)	A: self-practice (once weekly 2–5) (distributed) B: self-practice (four consecutive days in week 1 or 2 or 3 or 4 or 5 or 6) (massed) (Classes) (TOT 10 min sessions) (no feedback)	Practical assessment (week 6) (NGR) (not identical)
(Fendos, 2020) Phase 4	Anatomy (course)	A: REC and CR (weekly class) (feedback) ^α^ B: normal class (No TOT recorded)	REC and CR (midterm and final exam) (SA, grade total A: 60% B: 80%) (identical)
(Francis et al., 2020)	Introductory psychology (course)	A: FR concept map (4 topics in class) (feedback) ^α^ B: REC (4 topics in class) (feedback) C: no intervention (2 topics in class) (Approximate TOT similar A & B)	REC (week 4, 8, 12 and 16) (SA) (not identical, similar)
(Glass et al., 2013)	Psychology (course)	A: Included REC 21 questions identical to unit exams ^α^ B: Did not include REC 21 questions identical to unit exams (Final Exam) (no feedback reported)	REC (42 questions) (month 4 post final exam) (NGR) (identical)
(Gopalan et al., 2020)	Biology of cardiovascular and metabolic diseases (course)	A: Closed-book individual quiz and team-based recall (weekly class) B: Open-book individual quiz and team-based learning (weekly class) (Retrieval type not reported) (feedback) (no TOT recorded) (question type not reported)	REC and CR (approximately week 4, 8 and 12) (SA) (not reported if identical)
(Gurung & Burns, 2019)	Introductory psychology (course)	A: REC (more distributed, required once ^α^ B: REC (more distributed, required multiple times C: REC (less distributed, required once ^α^ D: REC (less distributed, required multiple times (Varying TOT) (mostly feedback) (SA – 10–34%)	REC (varying retrieval interval) (SA – 30–73%) (mostly non-identical)
(Hernick, 2015)	Pharmacology (2-month course)	A: REC (access online, unlimited attempts, 6 modules, no TOT recorded) (feedback) ^α^ B: Historical control no modules	REC (no retrieval interval reported) (SA) (partially identical)
(Higham et al., 2022)	Introductory psychology (course)	A: CR (feedback) (successive relearning) ^α^ B: Reread (Weeks 2–11, each week’s topic repeated 3x that week) (Online homework, participation 0.5% per learning session) (ToT A < B)	CR (week 12 and 15) (FA – participation 2.5% per exam) (1/3 identical, 1/3 transfer, 1/3 new)
(Iwamoto et al., 2017)	Introductory psychology (course)	A: REC (feedback) ^α^ B: Discussion and summary (Classes, 10 min each class)	REC (no retrieval interval reported) (SA, high stakes) (not identical, similar)
(Janes et al., 2020) Experiment 1	Neuroanatomy and neurophysiology (portion of course)	A: CR successive relearning (assigned homework, topic 1 days 3, 6 and 10, topic 2 days 12, 15 and 18) (feedback) ^α^ B: CR (optional homework) (feedback) C: Content no CR provided D: Historical control no CR (No TOT recorded)	REC (day 19) (SA, high stakes) (not identical)
Experiment 2	Neuroanatomy and neurophysiology (portion of course)	A: CR successive relearning (assigned homework, topic 1 days 3, 7, 10, topic 2 days 12, 15, 18) (feedback) ^α^ B: CR (optional homework) (feedback) C: Content no CR provided D: Historical control no CR (No TOT recorded)	REC (day 21) (SA, high stakes) (not identical)
(Kerdijk et al., 2015)	Internal medicine (course)	A: REC (cumulative exams weeks 4, 8, 10) B: REC (cumulative exam week 10) (Self-study TOT self-reported, highest in A)	A&B: REC in week 10 (SA, high stakes) (48 identical)
(Keus et al., 2019)	Cell biology and biochemistry (course)	A: Two midterm exams ^α^ B: One midterm exam (No TOT recorded) (feedback not reported) (question type not reported)	Cumulative final exam (SA – A: 35% B: 30%) (question type not reported, or if identical)
(LaDisa & Biesboer, 2017)	Pharmacotherapy (portion of course)	A: REC and CR (weekly homework, student generated and shared, no TOT recorded) ^α^ B: Historical control (no generation)	REC and CR (unit exam week 5) and REC (final exam week 16) (NGR) (not identical)
(Lawson, 2022)	Introductory psychology (course)	A: CR ^α^ B: REC ^α^ C: No Quiz (No TOT recorded) (open book) (feedback) (1% for participation in each of 8 quizzes)	REC (4 unit exams, no retrieval interval reported) (NGR) (20–30% identical)
(Linderholm et al., 2016)	Exercise physiology (class)	A: Read, FR, read ^α^ B: Read, read, read (Class) (TOT 3 × 2 min)	CR (day 7) (FA) (not identical)
(Logan et al., 1975)	Psycho-motor dentistry skills (course)	A: Practice sessions (TOT 6 × 1 h) B: Practice sessions (TOT 3 × 2 h) C: Practice sessions (TOT 2 × 3 h) (Classes) (no feedback reported)	Practical exam (no retrieval interval reported) (NGR) (not identical)
(Messineo et al., 2015)	General psychology (course)	A: REC (homework weekly) (feedback, limited TOT) ^α^ B: Reread (homework weekly) (no TOT recorded)	REC (week 10) (NGR) (not identical, similar)
(Miller & Srimaneerungroj, 2022)	Introductory psychology (course)	A: REC (frequently incorrect questions post 4x unit exams, SA – 0–10%) (feedback) ^α^ B: No restudy	REC (days post each unit exam − 99, 71, 40, 12) (NGR) (identical)
(Moore & Chalk, 2012)	Muscle stretch reflex (portion of degree)	A: Practical class and test (first and second year) ^α^ B: Theory class (first year), practical class and test (second year) (No TOT recorded) (no feedback reported)	Practical exam (second year) (NGR) (identical)
(Nevid et al., 2016)	Introductory psychology (class)	A: FR open book B: FR closed book (no feedback) C: CR open book D: CR closed book (no feedback) (TOT 15 min) (lab/class)	REC (day 7) (NGR) (not identical)
(Oermann et al., 2022a, b)	Cardiopulmonary resuscitation (classes)	A: Practice (4 sessions, 4 consecutive days) B: Practice (4 sessions, 4 consecutive weeks) C: Practice (4 sessions, 4 consecutive months) D: Practice (4 sessions, 4 consecutive yearly quarters) (No TOT reported) (feedback)	Practical exam (3 or 6 months) (NGR) (identical)
(Oermann et al., 2022a, b)	Cardiopulmonary resuscitation (classes)	A: Practice (3 months) ^α^ B: Practice (6 months) C: Practice (personalised interval) ^α^ (Distributed over 1 year, no TOT reported) (feedback)	Practical exam (1 year) (NGR) (identical)
(Opre et al., 2022)	Introductory psychology (course)	A: REC (student generated) (feedback) B: Normal study (No TOT reported)	REC (no retrieval interval reported) (NGR) (not identical)
(Osterhage et al., 2019) Study 2	Introductory biology (course)	A: Demonstrating overestimation in JOL and education on retrieval practice (classes, no TOT recorded) ^α^ B: No demonstration and education	REC (no retrieval interval reported) (SA)
(Palmen et al., 2015)	Anatomy (intensive course)	A: REC (optional online daily, weeks 1–3) (feedback) B: REC (optional online weekly, weeks 1–3) (feedback) C: No REC provided (No TOT recorded, unlimited attempts)	Question type not reported, or if identical (week 4) (SA)
(Palmer et al., 2019)	Pharmacy (class)	A: CR (1 week after lecture) (no feedback reported) (TOT mean 35 min) B: Rewatch lecture (1 week after lecture) (TOT mean 12 min)	REC and CR (day 7) (NGR) (not reported if identical)
(Poorthuis & van Dijk, 2021)	Psychology assessment in youth (course)	A: REC and CR (weekly homework) (feedback) (Graded 0.1% if > 70% correct, no TOT recorded) ^α^ B: Lecture (class weekly, 2 h, low attendance)	REC (week 4, 10) (SA – total 50%) (not reported if identical)
(Schmidmaier et al., 2011)	Clinical nephrology (class)	A: CR (No feedback) B: Restudy (Class successive relearning x4) (similar TOT)	CR (month 6) (NGR) (identical)
(Schneider et al., 2019)	Biochemistry (portion of course)	A: Video and REC (no feedback) ^α^ B: Video and restudy C: Video only (Class) (self-reported TOT higher for B)	REC (week 1.5) (NGR) (not identical, similar)
(Sennhenn-Kirchner et al., 2018)	Dental surgical suturing (course)	A: Practical assessment (month 5 and 6) (feedback) ^α^ B: Practice (month 5 and 6) (feedback) (Class) (TOT 10 min)	Practical assessment (month 7) (SA) (identical)
(Shobe, 2022)	Introductory psychology (course)	A: REC (group response in class 2/week, approximately 2 weeks per unit) (feedback) (TOT: 8–15 min) ^α^ B: Normal class	REC (6x unit quizzes post unit content) (SA) (50% identical)
(Terenyi et al., 2018)	Pharmaceuticals (course)	A: Study (week 1 or 3) CR (week 2 and 10 or 4 and 11), contracting distribution ^α^ B: Study (week 3 or 5) CR (week 4 and 8 or 6 and 9), equal distribution C: Study (week 1 or 4) CR (week 2 and 5 or 5 and 8), expanding distribution D: Study (week 2 or 5) CR (week 3 or 6), massed E: Study only (week 2 or 4) (Homework) (no feedback reported) (TOT 15 min limit on quiz, no TOT study)	CR (week 12 exam, week 18 long-term retention) (NGR) (not identical, similar)
(Terenyi et al., 2019) Experiment 1	Pharmaceuticals (course)	A: CR (weeks 6–10), equal distribution B: CR (weeks 5–10), expanding distribution ^α^ C: CR (weeks 5–10), contracting distribution (Homework) (no feedback reported) (TOT 15-25 min limit depending on quiz size, no TOT study)	CR (week 12 exam, week 18 long-term retention) (NGR) (not identical, similar)
Experiment 2	Pharmaceuticals (course)	A: CR assisted (fill in letters) B: CR unassisted (fill in whole word) ^α^ (Homework) (no feedback reported) (TOT 15-25 min limit depending on quiz size, no TOT study)	CR (week 12 exam, week 16 long-term retention) (NGR) (not identical, similar)
(Timmer et al., 2020)	Vaccination (class)	A: Lecture (15 min content and 5 min distractor x3, spaced) B: Lecture (7.5 min distractor 45 min content 7.5 min distractor, massed)	CR (day 8) (FA)
(Trumbo et al., 2016)	Introductory psychology (course)	A: REC (required weekly, 45% final grade) (TOT mean 7min26sec) ^α^ B: REC (optional weekly, no grade) (TOT mean 1min22sec) (Unlimited attempts) (feedback)	REC (3 unit exams one final exam) (SA total A: 50% B: 94%) (identical, similar)
Experiment 2	Introductory psychology (course)	A: REC (weekly homework) (TOT measured) ^α^ B: Control (no provided REC) (SA total 33%, unlimited attempts) (feedback)	REC (11 weekly unit exams (SA total 50%) (not identical, similar)
Experiment 3	Introductory psychology (course)	A: REC (weekly homework) ^α^ B: Reread (weekly homework) (SA total 33%, unlimited attempts) (no TOT recorded) (feedback)	REC (11 weekly unit exams (SA total 50%) (not identical, similar)
(Wong, 2022)	Pathology (course)	A: CR (class ‘think-pair-share’) (no TOT) (feedback) B: Normal class	Retrieval type not reported (retrieval interval not reported) (SA)

Note. Positive effect of an intervention is denoted with ^α^. Recognition (REC); Cued recall (CR); Short answer question (SAQ); Fill-in-the-blank (FIB); Free recall (FR); Summative assessment (SA); Formative assessment (FA); No grade reported (NGR); Time on task (TOT); Judgement of learning (JOL).

### Summary of articles

Of the 56 studies, some studies conducted more than one experimental intervention. Therefore, a total of 63 experiments are included in this review. Of these experiments, 43 demonstrated a significant positive effect for distributed practice and/or retrieval practice compared to massed practice, rereading, normal class, or no intervention. One study demonstrated a negative effect for distributed practice. Retrieval practice alone was most studied (n = 33), the spacing out of retrieval practice was commonly studied (n = 16) and distributed practice alone was less frequently studied (n = 14).

The most common units were introductory psychology (n = 16), physiology (n = 8), anatomy (n = 6) and anatomy & physiology (n = 4). Interventions were generally classroom based rather than homework based. The content from one class only was assessed in fifteen studies and the retrieval interval reported for these studies was generally seven days (five days for two studies). Many other studies were longer, covering content of an entire unit, the retrieval interval based on the final exam. Nine studies included an assessment of knowledge post unit completion. Interventions and comparison groups varied widely.

Recognition or cued recall were the most common retrieval types with only a few studies using free recall. Four studies compared types of retrieval practice and predominantly found fill-in-the-blank words more effective than fill-in-the-blank letters, short answer questions more effective than fill-in-the-blank, and free recall more effective than recognition. Feedback was common in retrieval practice interventions, however some studies failed to report on this at all.

Of the distributed practice studies, five compared types of distributed practice and found an expanding schedule more effective than an equal schedule in two studies but no difference in one study, an expanding schedule more effective than contracting and equal schedules in one study, and a contracting schedule more effective than expanding and equal schedules in another study. An expanding schedule was superior in three out of the five studies.

Time on task was frequently not reported, those studies that did measured time on task often did not control for this variable. Assessments were most frequently summative (n = 24) compared to formative (n = 14). Studies that used a small grade, for example, 2%, as incentive to complete the assigned work and was independent of final assessment outcomes, were included as formative assessment. Notably, many studies did not report on the stakes of assessments at all (n = 25), nor what percentage grade was assigned to the assessment. Two studies compared assigned versus optional homework and found assigned homework to be more effective (Janes et al., 2020; Trumbo et al., 2016). Five summative and two formative assessments showed no effectiveness of distributed practice and/or retrieval practice.

### Study quality

Of the studies that completed multiple experiments, those that used the same methodology in either the MERSQI or NOS-E are only rated once, however, if the methodology differed, they were rated separately under the relevant experiment. Within-subject studies were considered randomised if the order of interventions was randomised so that time until assessment was averaged over the conditions. The between-subject studies most often used the same community to select their comparison group, and historical cohorts were generally described as having the same class structure and content. Some studies did not mention or require ethical approval, and therefore did not include informed consent proceeding their randomisation. This resulted in a few of these studies not reporting any allocation concealment, and therefore scoring lower on the NOS-E. Non-randomised studies occasionally recorded subject characteristics, such as gender, age, and ethnicity, but infrequently recorded baseline scores such as a pre-test and rarely controlled for these characteristics with a statistical covariate analysis resulting in a lower comparability of groups.

Most studies were only sampled from a single institution. The retention of participants, which is scored in both the MERSQI and NOS-E was generally high. The representativeness of the intervention group was scored low in some studies, most often because the numbers were not reported. Blinding of assessment was scored high on most studies in the NOS-E, as most assessments were multiple-choice questions. No experiments included outcomes of ‘Behaviours’ or ‘Patient or healthcare outcomes’.

On the MERSQI, the ‘Validity evidence for evaluation instrument’ was generally scored low, with only two studies reporting on internal structure demonstrating reliability. Many studies did not report the source of content for their assessment, whether from a textbook, or expert. The data analysis was generally sophisticated and appropriate for all but a couple of studies.

## Discussion

This systematic review was undertaken to determine the effect of distributed practice and retrieval practice on academic grades in health professions education and to summarise a range of interventional variables that may affect study outcomes. This review indicates that distributed practice and/or retrieval practice are effective in most of the studies, when compared to several comparison or control groups, at improving test and examination scores, and is therefore a worthwhile learning strategy to consider in health professions education. Only one study showed a negative effect for distributed practice, but several variables such as time on task and test delay may explain this result (Cecilio-Fernandes et al., 2018). Although retrieval practice was the most studied learning strategy, many retrieval practice studies did not report on what content was being reviewed. Therefore the number of spacing out of retrieval practice interventions may be underreported if content from numerous weeks was being reviewed.

Interventions were generally applied to specific units of learning, rather than entire programs or content matter. Studies of these introductory units did not always include clear healthcare program information; however, it was assumed that these introductory units are a requirement for many health professions programs.

Not reporting the time on task for intervention and comparison groups was common in this review and is a strong confounding factor limiting the strength of the results. A false positive may occur when there is more time on task or a false negative when there is less time on task. One study reported a higher time on task for the distributed condition compared to the massed condition, but still found no significant benefit (Kerdijk et al., 2015). In this case, having students distribute their practice would be particularly ineffective, and a poor use of time. Whereas another study showed a higher time on task for the restudy group compared to the retrieval group, and even though they spent less time studying, the retrieval group had significantly superior outcomes (Schneider et al., 2019). This would then be considered a particularly effective learning strategy in this context, not to mention an efficient strategy. Conclusions may be erroneously drawn about the effectiveness of the learning strategies when time on task is not monitored and should be a focus of future research.

There are many variables mentioned in this review that could be affected by an overarching variable of student motivation. These include whether interventions were classroom-based or homework-based, optional homework or assigned homework, and summative or formative assessments.

Classroom-based interventions may result in students having less competing distractions and challenges with time management than in a home environment for homework-based interventions (Xu, 2013). Although only a couple of experiments mentioned online homework specifically, it is important to be aware that a face-to-face intervention compared to an online intervention may affect learning. Many health professions programs changed to elements of online learning during and post COVID (Kumar et al., 2021; Naciri et al., 2021; Schmutz et al., 2021). Results are mixed as to which may be more effective for student, but issues around student motivation, engagement and academic integrity may be relevant (Miller & Young-Jones, 2012; Platt et al., 2014). This will be an area to watch as more studies report on the effect of online learning compared to face-to-face learning in general, as well as with distributed practice and retrieval practice.

The two studies that found assigned homework to be more effective than optional homework may also be affected by student motivation (Janes et al., 2020; Trumbo et al., 2016). Assigning compulsory homework is an external motivation that will likely rise to the top of a student’s priority list compared to optional homework. To better understand the effect of motivation in these studies, measuring time on task for each group would give further insight. One study did not measure time on task (Janes et al., 2020), and the other found that on average, the more effective assigned homework group completed approximately 5.5 times the amount of time on task compared to the optional homework group. Therefore, although assigned homework is more effective, this is likely due to the student’s motivation to complete it and spend more time on the task.

There was no clear benefit in this review when comparing summative and formative assessments. It was a common area of missing information in the experiments and the study methodologies varied significantly. Further research should include this information and the percentage of the grade, as well as other factors that may explain the variability in outcomes, such as time spent studying and test anxiety.

There were also three interventions that used the placement of exams as distributed retrieval practice. Considering that most exams are a requirement to complete a unit and progress through a degree, this would be considered a high external motivation to increase effort to study and recall information, and therefore improve the retrieval practice effect. Two of these studies assessed increasing the number of exams, with one showing no significant benefit (Kerdijk et al., 2015) and the other showing that increasing the number of exams was a significant benefit (Keus et al., 2019). The time on task was not reported for either study, however it likely increased as the number of assessments increased. The time that students spent studying for each exam was also not reported, and likely increased due to the external motivation of a summative assessment. The third study looked at a post final exam assessment of knowledge retention and found that including content in the final exam increased the likelihood of improved long-term knowledge retention (Glass et al., 2013). This study also highlights the pitfalls of assessments in general, as many students may cram for an assessment and show positive outcomes but forget the knowledge in the long term. Overcoming this challenge may involve future research including more long-term, post-unit formative assessments that students don’t necessarily know about in advance, to get a clear gauge on the effectiveness of different learning strategies.

Student motivation is a complex, multifactorial topic and is not heavily addressed in this paper. It was not considered by any of the interventional experiments included in this review either. However, it may be an important variable to consider in future research, as students that participate in an intervention will of course benefit from these learning strategies more than students that chose not to participate, or only partially participate.

Study duration was another factor that may affect replication in ‘real world’ classrooms. Many studies only had a single intervention point which was then assessed a week later. This is frequently reported in prior research (Karpicke & Blunt, 2011; McDaniel et al., 2009) and research demonstrates that forgetting new content may plateau by one week (Loftus, 1985). The longer duration studies, however, may be more relevant to educators’ goals of long term memory in health professions education. This long term memory is needed for the scaffolding of information in future units (Belland et al., 2017) and to ultimately be applied in students professions post-graduation. Interventions and comparison groups varied widely, including studies that educated students on the benefits of distributed practice and/or retrieval practice as the intervention (n = 5). The goal of this type of intervention may be to improve students own self-directed studying. Considering educators limited time (Inclan & Meyer, 2020) and students reporting low use of these strategies in previous research (Persky & Hudson, 2016), this could be an interesting direction for future research. This future research should aim to students’ self-directed learning durations and type, as well as academic scores, to best understand if this is an effective strategy or not.

This paper supports the theory that increasing cognitive demand improves outcomes (Adesope et al., 2017), with short answer and free recall questions showing greater benefit than recognition questions. An expanding schedule was the most effective inter-study interval, however study methodology such as retrieval interval differed between studies and the results are mixed. Further research is needed to determine the most effective inter-study interval. There was insufficient information to determine if providing feedback with retrieval practice was more effective than not providing feedback, as there were no comparison groups directly assessing this, and most studies either provided feedback, or did not report on it at all. Future research has many avenues to understand this further, including the timing of feedback, with delayed feedback showing superior outcomes on other research (Butler et al., 2007).

### Study quality

The MERSQI and NOS-E scores in this review were affected by the inclusion criteria, which avoided low scoring in the ‘Study design’, ‘Type of data’ and ‘Outcome’ sections. (Cook & Reed, 2015). Between-subject studies were most commonly unable to be randomised when comparison groups were entire classes. A benefit of the within-subject design is an identical population comparison group, which therefore scored well in the NOS-E.

Most studies will have limited the generalisability of their results considering they were only sampled from a single institution. However, this is also simpler for researchers to control for variables such as content delivered and assessed. Considering that no two units are the same, a complete replication of these findings in future research or in educators’ classrooms cannot be expected, however it does still provide some good direction for future application of these strategies.

Studies that did not receive a full score for participant retention were generally large class sizes of an introductory unit, which often have higher student attrition (Trumbo et al., 2016) or studies that were of a longer duration (Dobson, 2013). High participant retention was noted in interventions that only addressed a single class, most likely due to the short duration of the study (one week). Incentives such as small amounts of course credit or money also likely improved retention in other studies.

No experiments had outcomes that included ‘Behaviours’ or ‘Patient or healthcare outcomes’, likely due to the nature of undergraduate education studies. Undergraduate health professions education does not generally apply learning to a patient population in a clinical context, compared to graduate training and continuing professional development (Chen et al., 2004). However, even in continuing professional development environments, assessing learning outcomes via patient or healthcare outcomes is poorly researched (Chen et al., 2004; Prystowsky & Bordage, 2001).

The low ‘validity evidence for evaluation instrument’ found in most studies, will limit the ability to generalise these outcomes into other settings, such as future high stakes examinations and professional practice (Beckman et al., 2005). Many studies did not report the source of content for their assessment, whether from a textbook, or expert. This domain of the MERSQI is commonly scored poorly in previous research (Reed et al., 2007). The time and resources required to validate an assessment is likely the cause of this finding, however future research should report on the source of content for assessment where possible. The high score of data analysis in this review is a domain that is typically scored high in other research (Reed et al., 2007).

### Recommendations

Based on this systematic review, educators and students may find distributed practice and retrieval practice effective in their own classroom or self-directed study context at improving academic grades. Foundational units, such as introductory psychology, anatomy, and physiology, could particularly benefit from these learning strategies. Educators could trial increasing the number of formative and summative assessments as a method of providing students with retrieval practice and distributed practice. This may improve academic grades and long-term memory of content for future units and professional practice. Expanding the distributed practice schedule may provide greater benefits compared to equal or contracting schedules. Free-recall or cued recall questions are likely to improve learning more than recognition questions such as multiple choice. Educators could also educate students on the benefits and practical applications of distributed practice and retrieval practice, which may improve self-regulated learning. Students could be encouraged to trial spacing out their revision, writing their own retrieval questions or sourcing questions from peers and external sources to improve their academic grades and long-term memory.

### Limitations

Due to the heterogeneity of studies, a meta-analysis is not possible. A single reviewer scored the MERSQI and NOS-E of the included papers and this may increase the risk of errors. The study quality instruments of both the MERSQI and NOS-E do not cover all aspects of study quality; elements that were missing include trial registration, which aims to reduce many types of bias, such as citation bias (Pannucci & Wilkins, 2010) and inflation bias or ‘p hacking’ (Head et al., 2015). Summarising the key variables and statistical significance from each study is also simplistic and can therefore be misleading if read in isolation. This review should be used to help navigate readers to source articles for the full picture, and not be used as evidence alone of a study’s significance.

## Conclusion

Distributed practice and retrieval practice are often effective at improving academic grades in health professions education. Of the 63 experiments, 43 demonstrated significant benefits of distributed practice and/or retrieval practice over control and comparison groups. Study quality was generally good with an average of 12.23 out of 18 on the MERSQI and an average of 4.55 out of 6 on the NOS. Key areas of study quality improvement are the validity of assessment instruments and the number of included institutions within a study. Future studies should consider measuring and reporting time on task which may illuminate the efficiency of distributed practice and retrieval practice. The stakes of the assessments, which may affect student motivation and therefore outcomes, should also be considered. Educators can note that the use of multiple exams, particularly if they are summative, will result in most students participating in the spacing out of retrieval practice. Introductory psychology, anatomy and physiology educators and students have a variety of retrieval practice and distributed practice applications that could be trialled and may successfully transfer to their own contexts.
